# Assessment of myocardial abnormalities in rheumatoid arthritis using a comprehensive cardiac magnetic resonance approach: a pilot study

**DOI:** 10.1186/ar3131

**Published:** 2010-09-13

**Authors:** Yasuyuki Kobayashi, Jon T Giles, Masaharu Hirano, Isamu Yokoe, Yasuo Nakajima, Joan M Bathon, Joao AC Lima, Hitomi Kobayashi

**Affiliations:** 1 Department of Radiology, St. Marianna University School of Medicine, 2-16-1 Sugao, Miyamae-ku, Kawasaki, Kanagawa, 216-8511, Japan; 2 Division of Rheumatology, Johns Hopkins University School of Medicine, Mason F. Lord Building-Center Tower, Suite 4100, 5200 Eastern Avenue, Baltimore, MD 21224, USA; 3 Division of Cardiology, Tokyo Medical University, 6-7-1 Nishi-shinjyuku, Shinjyuku-ku, 160-0023, Tokyo, Japan; 4 Division of Rheumatology, Internal Medicine, Itabashi Chuo Medical Center, 2-12-7 Azusawa, Itabashi-ku, Tokyo, 174-0051, Japan; 5 Division of Cardiology, Johns Hopkins University School of Medicine, 600 N. Wolfe Street, Blalock 524, Baltimore, MD 21287, USA

## Abstract

**Introduction:**

Rheumatoid arthritis (RA) is a multi-organ inflammatory disorder associated with high cardiovascular morbidity and mortality. We sought to assess cardiac involvement using a comprehensive cardiac magnetic resonance imaging (cMRI) approach and to determine its association with disease characteristics in RA patients without symptomatic cardiac disease.

**Methods:**

RA patients with no history and/or clinical findings of systemic or pulmonary hypertension, coronary artery disease, severe valvular heart disease, atrial fibrillation, diabetes mellitus, or echocardiographic abnormalities underwent contrast-enhanced cMRI on a 1.5T scanner. Adenosine triphosphate was used to assess perfusion defects due to microvascular impairment or ischemia, and delayed enhanced imaging was obtained for the assessment of myocardial inflammation/fibrosis. We explored the associations of cMRI abnormalities with RA disease activity and severity measures.

**Results:**

Eighteen patients (78% female) with a mean age of 57 ± 10 years were studied. Eight patients (45%) demonstrated a myocardial abnormality. Perfusion defects under pharmacologic stress were seen in two patients (11%), one of whom had a circumferential subendocardial perfusion defect and one had a non-segmental subendocardial perfusion defect. Seven patients (39%) were found to have delayed enhancement, only one of whom also demonstrated a perfusion defect. Mean disease activity score (DAS)28 was significantly higher in the group with delayed enhancement compared to the group without by an average of 1.32 DAS28 units (4.77 vs. 3.44 units, respectively; *P *= 0.011). Corresponding trends to statistical significance were noted in systemic inflammatory markers, with both C-reactive protein (CRP) and erythrocyte sedimentation rate (ESR) quantitatively higher in the group with delayed enhancement. Other RA characteristics, such as disease duration, autoantibody status, and current treatments were not significantly associated with cardiac involvement.

**Conclusions:**

Myocardial abnormalities, as detected by cMRI, were frequent in RA patients without known cardiac disease. Abnormal cMRI findings were associated with higher RA disease activity, suggesting a role for inflammation in the pathogenesis of myocardial involvement in RA.

## Introduction

Rheumatoid arthritis (RA) is a multi-organ inflammatory disorder affecting approximately 1% of the adult general population. A reduction in life expectancy in RA patients is primarily due to an increase of cardiovascular events [1-4] associated with both ischemic heart disease and congestive heart failure [5,6]. Importantly, myocardial disease is typically clinically silent [7], only manifesting as myocardial dysfunction after an extended preclinical phase. Myocardial dysfunction may arise from a number of distinct processes, including micro- and macrovascular coronary ischemia, myocardial inflammation (myocarditis), and/or myocardial fibrosis [8], any of which may be active in RA. Histopathologic studies have confirmed an increased prevalence of each of these findings in RA [9-11], yet are limited by their source (autopsy) and age (most from the 1950s and 1960s).

Considering these issues, an updated appraisal of myocardial disease is warranted in RA, preferably using a technique that is non-invasive and can be serially followed over time for progression, potentially identifying patients at the greatest risk of cardiac-related morbidity and mortality. Although some non-invasive methods, such as conventional and tissue-doppler echocardiography and single photon emission CT (SPECT), may reveal functional abnormalities [12-16], spatial resolution is not sufficiently accurate to identify the range of potential myocardial abnormalities in RA. Contrast-enhanced cardiac magnetic resonance imaging (MRI) is a non-invasive tool for the diagnosis of ischemic and non-ischemic heart diseases. Contrast agents diffused to the interstitial space will be resorbed into the capillary bed and undergo renal excretion. When the myocardial tissue is damaged, the resorption rate of contrast will be diminished. Specifically, delayed myocardial enhancement (delayed enhancement: DE), particularly when there is a delay in contrast washout within the tissue can indicate myocardial inflammation, fibrosis or myocardial infarction (MI) [17-20]. Adding pharmacological stress can identify myocardial perfusion impairment in various forms of cardiomyopathy as well as ischemic heart disease. Areas of reduced perfusion within the myocardium (perfusion defects: PD) when pharmacologic stress is applied indicate vascular impairment. Segmental PDs (that is, corresponding to the distribution territory of an epicardial coronary artery) suggest macrovascular involvement, while diffuse or non-segmental PDs are highly suggestive of microvascular impairment [21,22]. Combining contrast enhanced MRI with pharmacologic stress can aid in identifying underlying pathophysiological features more accurately than each approach alone.

Recently, several investigators have reported on cardiac MRI for the assessment of myocardial abnormality in patients with systemic sclerosis [23-25] and systemic lupus erythematosus [26]; however, only one report of cardiac MRI has been published in RA [27]. In this case, cardiac MRI was used successfully to differentiate myocarditis from myocardial infarction [27]. We sought to identify myocardial abnormalities in RA patients using contrast enhanced cardiac MRI combined with pharmacologic stress perfusion. Further, we sought to explore the associations of RA disease-related characteristics with cardiac MRI abnormalities.

## Materials and methods

### Patients

Consecutive female and male patients with RA as defined by the American Rheumatism Association classification criteria [28] were recruited from the outpatient rheumatology clinic at Itabashi Chuo Medical Center between October 2007 and December 2008. Exclusion criteria were current or prior treatment with prednisone, pregnancy, evidence of cardiomegaly on chest X-ray, symptoms of heart failure, coronary artery disease (angina and/or electrocardiogram (ECG) signs of myocardial ischemia), systolic blood pressure < 90 or > 150 mm Hg, heart rate < 50 or > 130 bpm, pulmonary arterial hypertension (right ventricular systolic pressure > 40 mm Hg determined by echocardiography), severe valvular heart disease, atrial fibrillation, diabetes mellitus, hyperlipdemia, dyslipidemia, history of smoking, abnormalities in echocardiography, history of bronchoconstriction, contraindication to MRI, hypersensivity to gadolinium or adenosine triphoshate, and current or past treatment with prostacyclin. Informed consent was obtained from all patients and the study was approved by the ethics committee of Itabashi Chuo Medical Center, Tokyo, Japan.

### Covariate assessments

All patients completed questionnaires regarding medical history, and underwent clinical examination and basic screening for conventional atherosclerotic disease risk factors, including cigarette smoking, systolic and diastolic blood pressure measurement, chest X-ray, a 12-lead ECG and a standard echocardiogram at study entry. Routine laboratory assays including serum cholesterol, triglycerides, high-density lipoprotein (HDL), low-density lipoprotein (LDL) and fasting blood glucose concentration, were performed. Information on demographics, smoking, and family history was collected by questionnaire. Resting blood pressure was measured three times in the seated position, and the average of the last two measurements was used in the analysis. Hypertension was defined by systolic blood pressure > 140 mmHg, diastolic blood pressure > 90 mmHg, or antihypertensive medication use. Diabetes was defined as a fasting serum glucose > 126 mg/dl or use of antidiabetic medications. Hyperlipidemia was defined by serum cholesterol > 220 mg/dl, serum triglycerides > 150 mg/dl or LDL > 140 mg/dl. Dyslipidemia was defined by HDL > 40 mg/dl mg/dl. Composite CV risk measures were calculated using the Framingham hard 10-year CV risk (National Cholesterol Education Program) [29]. Plain chest radiographs were diagnosed by a single, trained radiologist blinded to patients' characteristics. ECGs were analyzed by a single, trained cardiologist blinded to patients' characteristics. Cardiac standard echocardiography, including 2D and Doppler echocardiogram, was performed by a trained technologist and diagnosed by a trained cardiologist blinded to patient characteristics. The person who analyzed the echocardiography knew nothing about the patient's clinical characteristics.

### RA-specific covariates

RA disease duration was calculated based on self-report from the time of physician diagnosis. Current and past use of glucocorticoids and of biologic and nonbiologic DMARDs was ascertained by interview. The rheumatoid factor was assessed by ELISA, with seropositivity defined at or above a level of 20 units/ml. RA activity was calculated using the Disease Activity Score for 28 joints with C-reactive protein (CRP) [30]. Anti-cyclic citrullinated peptide antibody was assessed by ELISA, with seropositivity defined at or above a level of six units per milliliter.

### MRI Scanning and Interpretation

Patients underwent cine MRI, pharmacological stress and rest perfusion, and delayed enhancement MRI. All contrast MR studies were performed with a 1.5T MRI scanner (Achieva, Philips, The Netherlands). Adenosine triphosphate was infused using an automated power injector at a constant rate of 0.16 mg/kg body weight per minute for five minutes as a pharmacological stress agent [31,32]. Subsequently, a gadolinium-based contrast agent (Magnevist, Schering AG, Berlin, Germany) was injected into the right antecubital vein (0.1 mmol/kg body weight) during a first-pass perfusion sequence using a IR balanced Turbo Field Echo sequence (TR 2.8 ms, TE 1.38 ms, FA 45, slice thickness 8 mm, preparation pulse delay 200 ms), and images in four contiguous short-axis orientations were acquired at every two heartbeats. Adenosine triphoshate infusion was stopped after completion of the sequence. After five minutes of stress perfusion MRI, rest first-pass perfusion was scanned by the same sequence and same injection protocol for the gadolinium contrast agent. After a 10-minute delay, delayed enhanced images were obtained by using 3D-Turbo Field Echo sequence (TR 5.1 ms, TE 2.5 ms, FA 15, slice thickness 8 mm).

**Figure 1:**
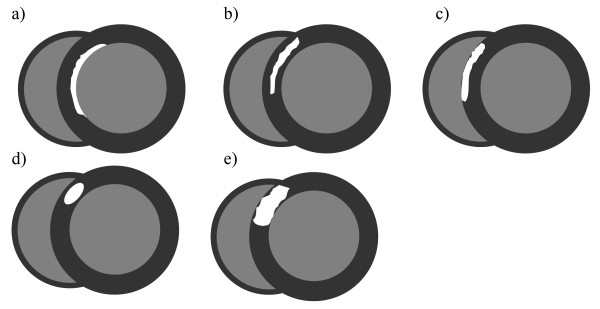
**Pattern of delayed enhancement**. **(a) **Linear enhancement in the subendocardial layer. **(b) **Linear enhancement in the middle layer. **(c) **Linear enhancement in in the subepicardial layer. **(d) **Nodular enhancement in the middle layer. **(e) **Patchy enhancement in the middle layer.

Images were viewed using VirtualPlace workstation (AZE, Tokyo, Japan) and independently analyzed by two radiologists and a cardiologist who were blinded to clinical information. Myocardial PDs detected during pharmacologic stress were categorized as segmental or non-segmental, based on whether they anatomically coincided with the distribution of an epicardial coronary artery. Myocardial DE lesions were characterized based on their presence, shape and anatomic location (Figure 1). Image enhancement was not characterized based on the intensity of enhancement. Table 1 summarizes the pathophysiologic interpretations used for different possible combinations of PD and DE lesions [33-35]. Non-segmental PD under stress with DE indicates microvascular impairment with myocarditis or fibrosis. Non-segmental PD under stress without DE indicates microvascular impairment without myocarditis and fibrosis. Segmental PD under stress without DE suggests macrovascular impairment without myocarditis and fibrosis, and segmental PD under stress with matched DE indicates myocardial infarction.

**Table 1:** Interpretation based on stress and rest perfusion and delayed enhancement MRI imaging

Stress perfusion	Rest perfusion	Delayed enhacement	Pathophysiological implication
Non-segmental PD (+)	PD (-)	DE (+)	Microvascular impairment with myocarditis or fibrosis
Non-segmental PD (+)	PD (-)	DE (-)	Microvascular impairment without myocarditis and fibrosis
Segmental PD (+)	PD (-)	DE (-)	Macrovascular impairment
Segmental PD(+)	Matched PD (+)	Matched DE (+)	Myocardial infarction
PD (-)	PD (-)	DE (+): Intermediate or Subepicardial layer	Myocarditis or fibrosis without micro- and macrovascular impairment
PD (-)	PD (-)	DE (+): Subendocardial layer	Focal myocardial infarction (fibrosis) without micro- and macrovascular impairment
PD (-)	PD (-)	DE (-)	Normal

PD, perfusion defect; DE, delayed enhancement; +, present; -, absent.

### Statistical analysis

The distributions of all variables were examined. In groups categorized by the presence of delayed enhancement, means and standard deviations were calculated for normally distributed continuous variables and compared using t-tests. Medians and interquartile ranges were calculated for non-normally distributed continuous variables and compared using the Kruskal-Wallis test. Counts and proportions were calculated for categorical variables and compared using Fisher's exact test. Multivariable linear regression was used to model the association of delayed enhancement with DAS28 score. Patient characteristics which differed by the presence of delayed enhancement (gender, age, RA duration, use of TNF inhibitors and Framingham 10-year hard cardiovascular disease (CVD) event risk score) were modeled as confounders. Because of the small sample size, hierarchical modeling was used with a maximum of three variables simultaneously included. The Shapiro-Wilk test confirmed normality of the untransformed DAS28 score across distribution of the modeled covariates. Statistical calculations were performed using Intercooled Stata 10 (StataCorp, College Station, TX, USA). In all tests, a two-tailed α of 0.05 was defined as the level of statistical significance.

## Results

Characteristics of the 18 participants are summarized in Table 2. Participants were mostly female (78%) with a mean age of 57 ± 10 years. RA duration ranged from 5 months to 16 years (median 2.7 years) with 10 participants (55.6%) categorized as having early RA (disease duration less than three years). While most (88.9%) participants were seropositive for rheumatoid factor (RF), only nine (50%) had anti-cyclic citrullinated peptide (CCP) antibodies. The mean DAS28 for the cohort was 3.96, with six participants (33.3%) falling into the low disease activity category (DAS28 < 3.2) and six participants (33.3%) falling into the high disease activity category (DAS28 > 5.1). TNF inhibitors were prescribed in seven (38.9%) of the participants. The median Framingham 10-year hard CVD event risk score was 2%. CV risk factors were generally low, consistent with the study design in which participants were deliberately selected based on having a low risk CV risk profile.

**Table 2:** Participant characteristics according to presence of delayed enhancement on cardiac magnetic resonance scanning

Characteristic *	Total *n *= 18	DE present *n *= 7	DE absent *n *= 11	*P*
Female, n (%)	14 (77.8)	7 (100.0)	7 (63.6)	0.12
Age, years	57.4 ± 10.3	61.9 ± 8.6	54.6 ± 10.6	0.13
SBP, mmHg	122 ± 14	119 ± 13	124 ± 14	0.48
DBP, mmHg	76 ± 9	71 ± 7	78 ± 9	0.09
Total cholesterol, mg/dL	179 ± 19	181 ± 19	178 ± 19	0.68
HDL-C, mg/dL	52 ± 9	51 ± 9	53 ± 9	0.56
LDL-C, mg/dL	100 ± 15	105 ± 18	97 ± 13	0.33
Triglycerides, mg/dL	134 ± 21	130 ± 26	137 ± 18	0.54
Ejection fraction (echocardiogram), %	66 ± 6	67 ± 6	66 ± 6	0.71
Any perfusion defects, n (%) **	2 (11.1)	1 (14.3)	1 (9.1)	1.00
RA duration, years; median (IQR)	2.7 (0.7 to 9.0)	8.8 (2.5 to 10.5)	1.6 (0.6 to 9)	0.26
RA duration < 3 year, n (%)	10 (55.6)	2 (20.0)	8 (80.0)	0.15
RF, n (%)	16 (88.9)	7 (100.0)	9 (81.8)	0.50
Anti-CCP, n (%)	9 (50.0)	2 (28.6)	7 (63.6)	0.34
DAS28; median (IQR)	3.96 ± 1.20	4.77 ± 0.78	3.44 ± 1.16	0.01
CRP, mg/L; median (IQR)	2.61 (1.10 to 4.22)	4.22 (3.08 to 5.28)	1.22 (0.50 to 3.40)	0.08
ESR, mm/hr; median (IQR)	47 (28 to 60)	60 (46 to 68)	39 (28 to 47)	0.09
HAQ (0-3)	0.88 (0.25 to 1.0)	1.0 (0.75 to 1.0)	0.25 (0.25 to 1.0)	0.28
Framingham risk score; median (IQR)	2.0 (0.5 to 4.0)	1.0 (0.5 to 6.0)	2.0 (1.0 to 3.0)	0.75
TNF inhibitor use, n (%)	7 (38.9)	1 (14.3)	6 (54.6)	0.15

* Characteristics are expressed as mean ± standard deviation unless otherwise noted.

** The two perfusion defects detected represented different processes. The subject with DE demonstrated a perfusion defect pattern consistent with microvascular impairment while the subject without DE demonstrated a perfusion defect pattern consistent with macrovascular impairment.

**Figure 2:**
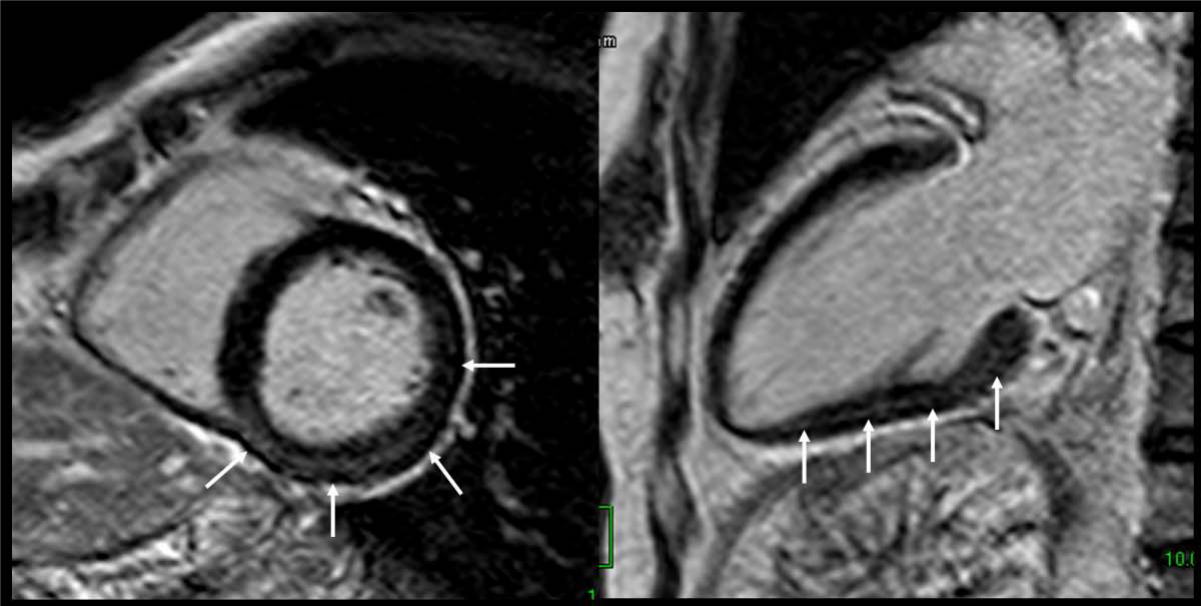
**Delayed enhancement of myocarditis**. Delayed enhanced images showed patchy enhancement in middle layer of inferior and lateral LV wall (arrows).

**Figure 3:**
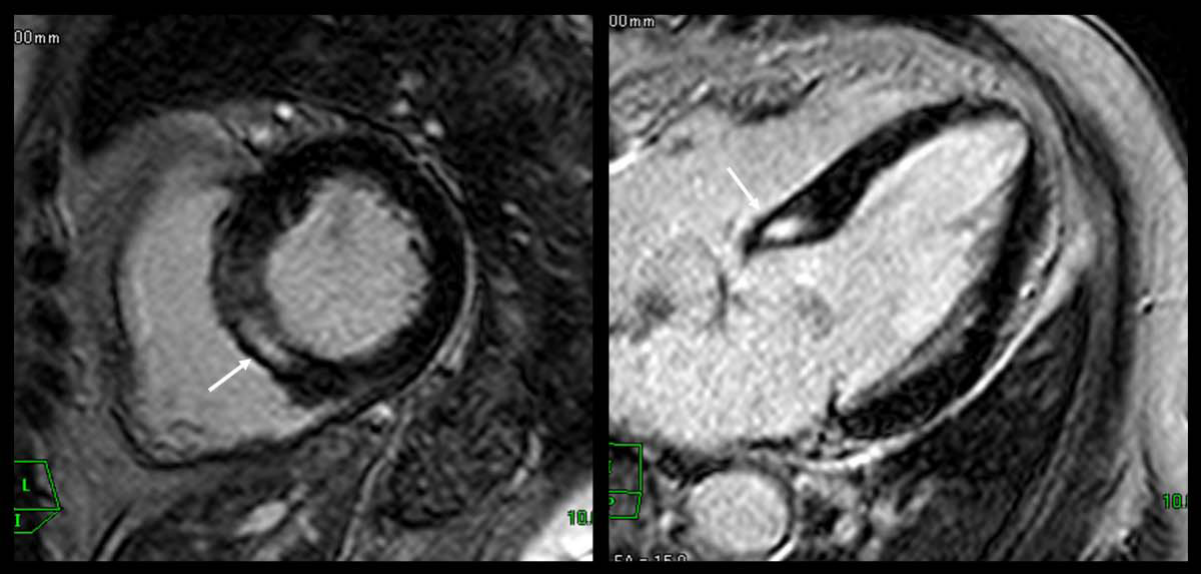
**Delayed enhancement of myocarditis**. Delayed enhanced images showed nodular enhancement in middle layer of septal wall (arrows).

**Figure 4:**
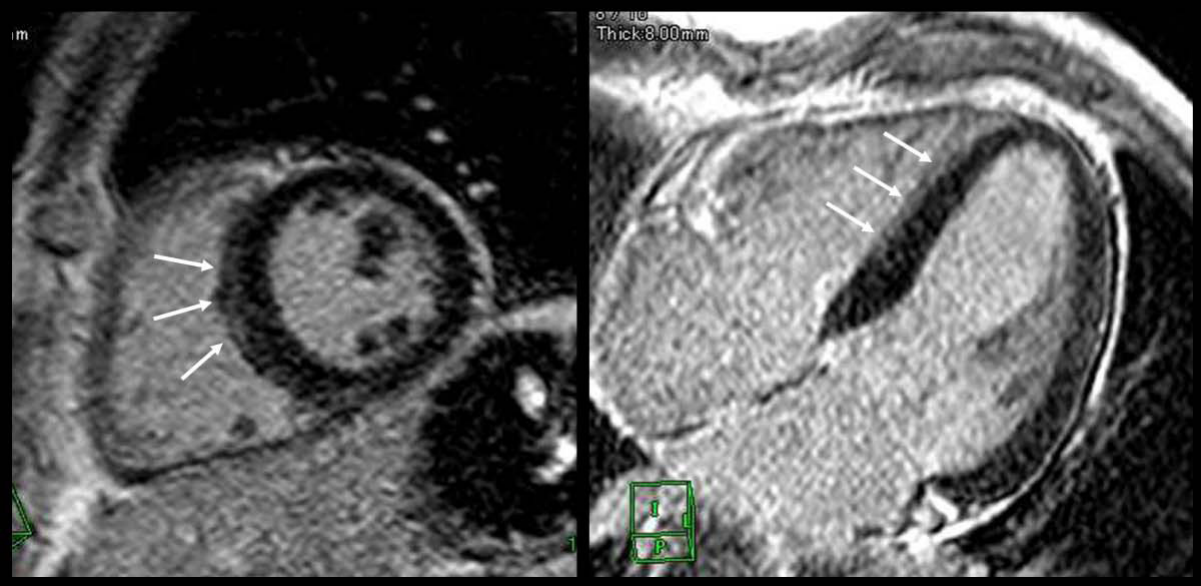
**Delayed enhancement of myocarditis**. Delayed enhanced images showed linear enhancement in subepicardial layer of septal wall (arrows).

Myocardial DE was observed in seven patients (38.9%). Among these, enhancement was observed in the middle layer in four patients (57.1%) (Figures 2 and 3), the subendocardial layer in two (28.6%), and the subepicardial layer in one patient (14.3%) (Figure 4). Patterns of enhancement included: patchy in four patients (57.1%) (Figure 2), nodular in two patients (28.6%) (Figure 3), and linear in one patient (14.3%) (Figure 4). Anatomically, enhancement was observed throughout the left-ventricle, occurring in the inferolateral wall (*n *= 2), lateral wall (*n *= 1), inferior wall (*n *= 1), anterior wall (*n *= 1), and in the septum (*n *= 1).

**Figure 5:**
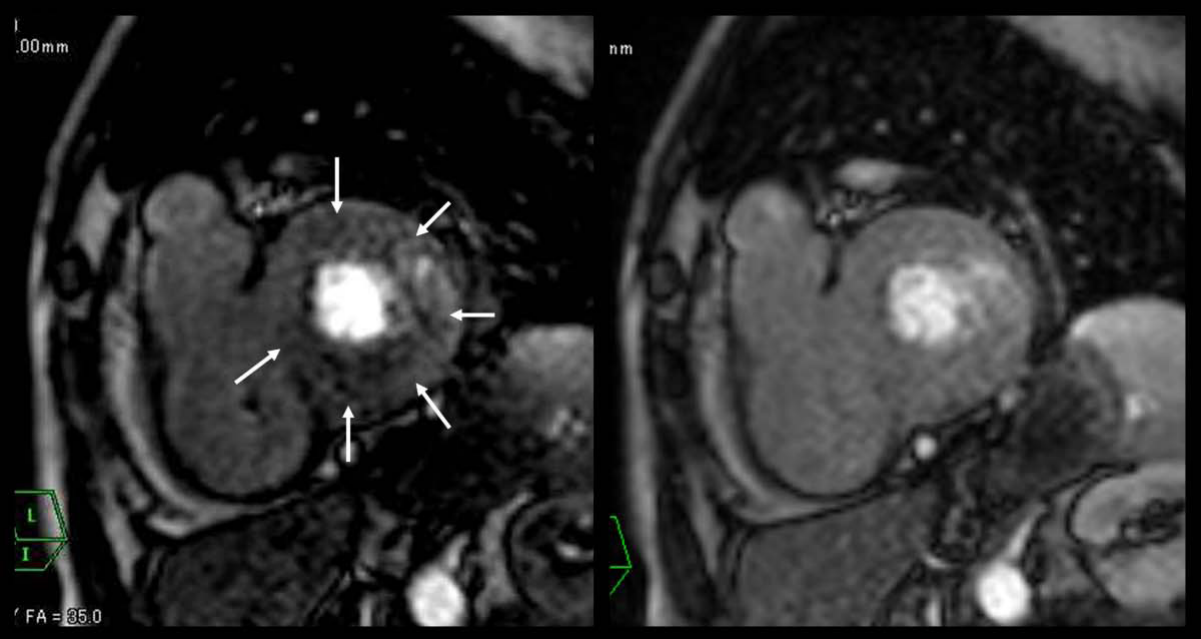
**Early perfusion defect of microvascular impairment without myocardial fibrosis or myocarditis**. Perfusion MRI under stress (left figure) showed non-segmental circumferential perfusion defect (arrows). Perfusion MRI at rest (right figure) showed no defects. In this case, delayed enhanced images showed no enhancement (not shown).

None of the participants demonstrated perfusion defects at rest. Perfusion defects under pharmacologic stress were observed in two patients (11.1%). In one (Figure 5), a circumferential patchy perfusion defect in the subendocardial layer was observed and was not associated with delayed enhancement, a pattern consistent with microvascular coronary artery disease without accompanying myocarditis or fibrosis. In the second participant with a perfusion defect under stress, the defect was non-segmental patchy in the subendocardial layer, a pattern consistent with microvascular impairment. This participant also demonstrated nodular delayed enhancement (Figure 3). Of note, microvascular impairment was not observed in the remaining six participants with delayed enhancement.

### Association of participant characteristics with myocardial delayed enhancement

Participant characteristics according to the presence of any abnormality of cardiac MRI and delayed enhancement are summarized in Table 2. Compared to patients without myocardial DE, patients with delayed enhancement were more likely to be female (100% vs. 64%, respectively) and were slightly older (mean age 62 vs. 55 years, respectively); however, none of these differences were statistically significant. Comparing patients with and without delayed enhancement, there were no clinically or statistically significant differences in current traditional cardiovascular risk factors, including echocardiographic parameters. Among RA parameters, median RA duration was greater, and the proportion of patients with early disease lower, in the group with delayed enhancement; however, these differences were not statistically significant. Likewise, the proportion of participants seropositive for RF or anti-CCP antibodies did not differ according to the presence of delayed enhancement.

Mean DAS28 was significantly higher in the group with delayed enhancement compared to the group without by an average of 1.32 DAS28 units (4.77 vs. 3.44 units, respectively; *P *= 0.011). Corresponding trends to statistical significance were noted in systemic inflammatory markers, with both CRP and erythrocyte sedimentation rate (ESR) quantitatively higher in the group with delayed enhancement. Despite higher disease activity overall, TNF inhibitors were prescribed in only one participant with delayed enhancement (14.3%) compared to six without delayed enhancement (54.6%); however, this difference was not statistically significant (*P *= 0.15). Delayed enhancement was seen in 63.6% of patients with negative anti-CCP-Antibody compared to 28.6% of those with a positive anti-CCP-Antibody (prevalence ratio 2.2), but this difference was not statistically significant (*P *= 0.34).

After adjusting for confounding by gender, the association of myocardial delayed enhancement with DAS28 was strengthened (Table 3, Model 2), in which the group with myocardial DE demonstrated DAS28 scores, on average, of 1.82 units higher than the group without myocardial DE. Additional adjustment for age, RA duration, TNF inhibitor use and Framingham 10-year hard CVD event risk score (Table 3, Models 3 to 6) did not modify the association of myocardial DE with DAS28 score. Myocardial DE and gender accounted for almost half of the total variability in DAS28 (R^2 ^= 0.49).

**Table 3:** Crude and adjusted associations of myocardial delayed enhancement with DAS28

Characteristic	Model 1		Model 2		Model 3		Model 4		Model 5		Model 6	
						
	β	*P*	β	*P*	β	*P*	β	*P*	β	*P*	β	*P*
Delayed enhancement*	1.32	0.018	1.82	0.002	1.89	0.004	1.78	0.003	1.71	0.005	1.88	0.002
Male gender*			1.36	0.031	1.39	0.036	1.48	0.036	1.47	0.029	1.78	0.042
Age, per year**					-0.01	0.74						
RA duration, per year**							0.02	0.64				
TNF inhibitor use*									-0.36	0.49		
Framingham risk score, per%											-0.07	0.45

R^2 (†)^	0.31		0.49		0.50		0.50		0.51		0.51	

* β coefficients represent the average change in the outcome (DAS28) for those with the characteristic vs. those without.

** β coefficients represent the average change in the outcome (DAS28) per 1 unit increase in the characteristic.

† R^2 ^is the percentage of the total variability in the outcome explained by the aggregate of the predictors in the model.

## Discussion

In this pilot study, we used cardiac MRI to assess myocardial abnormalities in RA patients with no cardiac symptoms. To our knowledge, this is the first investigation to combine both perfusion and delayed enhancement cardiac MRI for assessing cardiac involvement in asymptomatic patients with RA. Our preliminary data lend support for a low prevalence of microvascular impairment in the myocardium with RA patients, and a high prevalence of delayed enhancement, suggesting myocarditis or focal fibrosis. These findings have potential implications for research and clinical care. For research, they suggest that distinct pathologic processes affecting the myocardium may be identified and tracked in RA patients using a safe, non-invasive assessment tool, allowing for a more complete understanding of the RA disease process. For clinical care, early identification of myocardial disease may permit earlier intervention, potentially reducing the impact of myocardial dysfunction on cardiovascular morbidity and mortality in RA patients.

The clinical diagnosis of myocardial disease in RA remains problematic. Endomyocardial biopsy (EMB) may increase the frequency of detection; however, the sensitivity of EMB may be as low as 35%, primarily because of patchy involvement or focality of myocarditis [36]. Additionally, EMB carries a 1 in 250 risk of perforation and a 1 in 1,000 risk of death, even in experienced hands [37]. Therefore, less-invasive tools are needed to diagnose and track cardiac abnormalities. Recent advances in cardiac MRI enable dynamic first-pass perfusion MRI of the entire left-ventricular myocardium with improved image quality and higher spatial resolution compared to SPECT. Pharmacological stress perfusion MRI has been shown to be an accurate established method for detecting coronary artery disease (CAD) [38]. Fenchel *et al. *reported that stress examination increased the perfusion differences of MRI between normal and ischemic myocardial area compared with rest imaging [39]. Klem *et al. *[40] reported that the addition of delayed enhanced MRI to the stress-rest perfusion examination would help distinguish true perfusion defects from artifacts and improve test reliability to the point that rapid visual interpretation could be performed with high accuracy. Moreover combining stress perfusion and delayed enhanced MRI is an accurate method for characterizing pathologic features [33,35].

Other non-invasive modalities for myocardial imaging are available, but not as sensitive or versatile as MRI. Conventional echocardiography allows for estimation of global and regional myocardial function, but cannot distinguish specific etiologies of dysfunction with accuracy. In addition, echocardiography requires careful standardization, both for acquisition and interpretation, a limitation that is considerably less for MRI. For these reasons, in our study, echocardiography was performed solely to exclude patients with low ejection fraction and/or diastolic dysfunction and not compared to the cardiac MRI studies. SPECT has been used in two published papers in patients with RA [16,41]. Banks *et al. *reported, using adenosine stressed SPECT, that ischemic heart disease was twice as common and more often silent in 67 RA patients compared with 37 matched controls with osteoarthritis [41]. Noninvasive assessment of myocardial blood flow by adenosine stressed SPECT showed reversible ischemia and diffusely poor myocardial perfusion, suggesting myocardial microvascular disease [16]. Although myocardial perfusion SPECT is widely used in clinical practice and the high sensitivity of SPECT has been reported, the spatial resolution of this modality is limited and the radiation dose is problematic. Furthermore, Ishida *et al. *[42] reported that pharmacological stress perfusion MRI was superior to SPECT for the assessment of myocardial ischemia in the general population.

Non-segmental PD during pharmacological stress was seen in 2 out of 18 patients (11%). Non-segmental PD, not corresponding to any epicardial coronary artery distribution, is highly suggestive of microvascular impairment. Coronary arteritis is seen in about 20% of RA patients at autopsy [11]. Inflammation with edema of the intima of the artery may lead to severe narrowing or occlusion of its lumen, to necrosis, and angina or infarction [43]. Nevertheless, myocardial infarction secondary to coronary arteritis is rare in patients with RA [9]. In contrast to atherosclerosis, rheumatoid vasculitis frequently involves intramyocardial arteries, indicating microvascular impairment [9,11,44]. Raza *et al. *reported reversible ischemia and diffusely poor myocardial perfusion in one patient with RA using noninvasive assessment of myocardial blood flow by adenosine stressed SPECT [16]. Repeat assessment after intensive immunosuppression therapy revealed increased myocardial perfusion. Coronary angiography revealed no significant atheroma, suggesting that myocardial microvascular disease was responsible for the ischemia. Pharmacological stress perfusion MRI is a non-invasive tool to assess the microvascular circulation of myocardium [21,22]. And cardiac MRI can be repeatedly performed for the assessment of therapeutic effect or follow-up examination, because there is no radiation exposure. Our findings would support the prospective study of the effect of RA treatment, and associated change in joint disease activity, on myocardial perfusion defects.

Five patients (28%) had areas of DE in the middle or subepicardial layer of the left ventricle, suggesting myocarditis. The detection of DE, suggesting myocarditis or fibrosis, in 28% of our study participants is striking, particularly considering that all of the patients were asymptomatic, had no risk factors other than RA for these findings, and had normal conventional echocardiographic assessments. Myocarditis is a rare but recognized form of rheumatoid cardiac disease and is a known cause of congestive heart failure [45]. Rheumatoid myocarditis can assume a granulomatous form that is considered specific for RA, or a nonspecific form that may also be observed in other disorders. When present, the granulomas show a predilection for the left ventricle and are morphologically identical to the subcutaneous nodules of RA [9]. In contrast, the nonspecific inflammatory pattern involves the collagenous interstitium of the heart, and is composed predominantly of lymphocytes, plasma cells, and histiocytes [9,46]. In cardiac MRI, relatively greater gadolinium contrast enhancement in the myocardium has been reported in patients with active myocardial inflammation by histopathology [47]. There may be either focal or patchy diffuse enhancement of the left ventricular (LV) myocardium, typically with a subepicardial or midwall distribution [48-50]. However, isolated subendocardial distribution was not demonstrated. The LV lateral free wall is more often involved, followed by the ventricular septum [51]. Consistent with this, in our study, two out of five cases with myocarditis pattern had DE in the lateral free wall, and two had DE in the septum. In a study of individuals without RA, the extent of myocardial enhancement was roughly correlated with LV dysfunction, and it may decrease during healing and essentially disappear after recovery [47].

Two patients in our study (11%) had a subendocardial nodular DE, suggesting focal myocardial infarction. RA patients are less likely to report symptoms of angina and are more likely to experience unrecognized myocardial infarction and sudden death [52]. Interestingly, RA patients were more likely to be hospitalized for acute myocardial infarction or to have experienced unrecognized myocardial infarctions prior to their diagnosis of RA [52]. In myocardial infarction, cardiac MRI typically shows subendocardial DE [48,53]. In contrast, myocarditis shows a characteristic MRI pattern of contrast enhancement, which originates from the epicardium, sparing the subendocardial layer [53]. Our patients had no cardiac symptoms, no history of cardiac diseases, and normal ECG studies; therefore these DE findings may correspond to myocardial scarring from unrecognized myocardial infarctions. Barbier *et al. *[54] reported that 49 of 259 (19.8%) randomly chosen 70-year-old subjects had MI scars in delayed enhanced MRI. On the other hand, only one unrecognized myocardial infarction was found with delayed enhanced MRI in 298 subjects with a mean age of 50 years [55]. The higher frequency of findings suggesting myocardial scarring in our study compared to the above published report is compelling, but deserves study in a larger cohort of RA patients.

Interestingly, we detected a significant association between RA disease activity and inflammation and myocardial DE in cardiac MRI. In several autopsy studies of RA patients, those with myocarditis were noted to have active arthritis, endocarditis, or pericarditis and vascular changes, which strongly suggests that myocarditis is associated with active forms of RA [9-11]. Longitudinal follow-up is required to explore whether changes in disease activity are associated with corresponding changes in DE and the contribution of RA pharmacotherapies to dynamic changes in DE.

Our study had limitations. This was a pilot study and the size is too small for definitive conclusions. The lack of non-RA controls limits our ability to strongly conclude that our findings are unique to RA patients; however, the much higher frequency of myocardial abnormalities observed in the present study compared to studies conducted in non-RA patients with similar demographic characteristics and CVD risk factors is suggestive of a true disease effect. We did not perform T2-weighted MR images, which are more reliable in differentiating edema induced from acute myocarditis from fibrosis [56]. Nonetheless our preliminary data lend support for a high prevalence of myocarditis with asymptomatic RA patients.

## Conclusions

To the best of our knowledge, our study is the largest patient series to date to report cardiac abnormalities in asymptomatic patients with RA using pharmacological stress and rest perfusion, and delayed-enhanced MRI. The detection and histological confirmation of cardiac involvement in RA is often difficult because of a lack of symptoms and invasive diagnostic method such as EMB. Comprehensive cardiac MRI might be considered to be a useful and less-invasive diagnostic tool for assessing cardiac involvements in asymptomatic patients with RA. Further studies of this diagnostic tool should be evaluated to discuss its utility.

## Abbreviations

CAD: coronary artery disease; CCP: cyclic citrullinated peptide; cMRI: cardiac magnetic resonance imaging; CRP: C-reactive protein; CVD: cardiovascular disease; DAS: disease activity score; DE: delayed enhancement; ECG: electrocardiogram; EMB: endomyocardial biopsy; ESR: erythrocyte sedimentation rate; HDL: high-density lipoprotein; LDL: low-density lipoprotein; LV: left ventricular; PD: perfusion defects; RA: rheumatoid arthritis; RF: rheumatoid factor; SPECT: single photon emission CT.

## Competing interests

The authors declare that they have no competing interests.

## Authors' contributions

YK conceived of the study, participated in the design of the study and performed the statistical analysis. JG performed the statistical analysis and helped to draft the manuscript. HM participated in the coordination of the study and carried out MRI examinations. IY participated in the coordination of the study and patient management. YN and JL participated in the design of the study and in the sequence alignment. JB participated in the design of the study and helped to draft the manuscript. HK conceived of the study, participated in its design and coordination, and helped to draft the manuscript. All authors read and approved the final manuscript.
